# Effect of Materialism on Pro-environmental Behavior Among Youth in China: The Role of Nature Connectedness

**DOI:** 10.3389/fpsyg.2022.794816

**Published:** 2022-02-22

**Authors:** Jing Wang, Yongquan Huo

**Affiliations:** School of Psychology, Shaanxi Normal University, Xi'an, China

**Keywords:** materialistic values, pro-environmental behavior, nature connectedness, nature contact, mediation model

## Abstract

We designed three studies to explore the effect of materialistic values on pro-environmental behavior among youth and the mediated role of nature connectedness between materialistic values and pro-environmental behavior. Through a self-report questionnaire survey (Study 1) and an experimental manipulation of materialistic values (Study 2), we found that materialistic values negatively predicted pro-environmental behavior, and that nature connectedness played a mediating role. Further, we used natural contact strategies to control the level of nature connectedness, and found that the negative impact of high materialistic values on pro-environment behavior decreased with the increase of nature connectedness, further supporting the mediating role of nature connectedness (Study 3). These results may contribute to the design of strategies that effectively mitigate the negative effects of materialistic values on pro-environmental behavior.

## Introduction

In recent years, a variety of environmental problems have posed threats to environmental sustainability, including global warming, air pollution, water shortages, noise pollution, and the loss of biodiversity, many of which are caused by human activities and behavior (Vlek and Steg, 2010). Therefore, increasingly more attention has been paid to research on environmental protection behavior and its influencing factors in different fields (Rosa and Collado, 2019). Hines et al. (1987) suggested that such action to avoid or solve various environmental problems, known as pro-environmental behavior, is a conscious behavior based on personal responsibility and values. Recent research has found a close relationship between individual values and behaviors toward the environment (Hurst et al., 2013). Among the many factors that influence pro-environmental behavior, the important role of materialistic values has attracted attention in academic circles (Furchheim et al., 2020). Recent studies have shown that materialism is popular among Chinese consumers, especially young consumers (Ma et al., 2020). However, there is little research on the relationship between specific values and environment-friendly behaviors, especially in Eastern cultures such as China. Thus, it is important to gain a deeper understanding of the role of materialistic values in pro-environmental behavior among young consumers in the context of China, a developing Asian country where only modest research efforts have been made toward exploring this important topic.

## Materialistic Values and Pro-Environmental Behavior

Since China's reform and opening up, the urban economy has substantially developed, and the nation's urbanization process has advanced to gradually enter a “new normal” phase. In this process, China's population and industries continue to inundate its cities, and environmental pollution has become a critical problem (Hao et al., 2020). Materialistic values involves the increasing emphasis on material wealth as the center of life, source of happiness, and criterion for success (Richins and Dawson, 1992). In general, materialism is usually positively correlated with selfishness, self-centeredness, and extrinsic motivation (Shrum et al., 2013), and individuals with materialistic tendencies seem to pay more attention to externally visible characteristics and appearance than to social relationships. In particular, materialistic values can affect the development of society through their effects on individuals. According to the theory of value conflict, people's various values are not completely isolated, but coexist in an interrelated hierarchical structure (Schwartz, 1994). On these grounds, if individuals hold two competing or opposing values at the same time, they will experience a conflict of values. Specifically, concern for the health of our planet is reflected in self-transcendental values, which tends to be universalist and collectivist, while materialistic values are the opposite of these two values. There is evidence that a conflict between materialistic and green value profiles can arise in consumers (Furchheim et al., 2020), and it is illustrated that materialism is accompanied by less concern for ecological sustainability. Numerous studies have also found a significant negative correlation between materialism and pro-environmental behavior (see Hurst et al., 2013; Kasser, 2016). Increasingly, research in countries, such as Canada, Italy, and Sweden further supports this finding (Hirsh and Dolderman, 2007; Hultman et al., 2015; Lu et al., 2016). According to the above theory and the negative effects of material values, we hypothesize that: higher levels of materialistic value are associated with lower levels of pro-environmental behavior (H1).

## The Role of Nature Connectedness

Nature connectedness is a psychological connection to nature reflecting one's relationship with the natural environment (Mayer and Frantz, 2004; Tam, 2013). It means that the individual endows nature with human characteristics, perceiving nature as a feeling, thinking, and living object just like humans. Relevant studies show that materialism has been associated with lower quality relationships with friends and romantic partners (Kasser and Ryan, 2001). An individual's materialistic tendency may lead to higher indifference, lower empathy, and less attention to others' pain (Kasser, 2016). In addition, the interpersonal relationship (e.g., peers, parents, or teachers) can be extended to non-interpersonal relationships (e.g., the natural environment itself), in which individuals can undertake various commitments (Davis et al., 2009). A recent study showed that higher materialism was associated with a lower level of attachment to the places and people one lives in contact with, which was a stable predictor of one's level of pro-environmental behavior (Scannell and Gifford, 2013). These studies suggest that people with high materialistic values have lower levels of empathy and attachment to nature; that is, they have a weaker sense of connection with nature, and may thus further show a lower level of pro-environmental behavior.

In environmental psychology, the sense of nature connection may be a way to environmental sustainability (Zelenski and Nisbet, 2014). Individuals' sense of nature connection may motivate them to participate in pro-environmental behavior (Mayer and Frantz, 2004). Conversely, a lack of connection with nature has been blamed for individuals' indifference toward environmental degradation and protection (Pyle, 2003; Soga and Gaston, 2016). Roszak (1992), an American ecological psychologist, proposed the “ecological subconscious” concept in his book *The Voice of the Earth*. He believed that there is an innate and evolving emotional connection between humans and the environment. If the emotional connection is suppressed, individuals will not have a high sense of identity to fully correspond to the ecological environment, let alone treat themselves and nature equally. This will be detrimental to the development of an ecological self-concept and cognition as well as pro-environmental behavior. Studies have also shown that emotional attitudes toward nature can play a key driving role in individuals' actual behavior toward the environment (Pinho et al., 2014; Whitburn et al., 2020). Based on the above literature, it can be seen that there is a close relationship between materialistic values, pro-environmental behavior, and nature connectedness (Aruta, 2021). Specifically, activating individual materialistic values can lead to a decrease in the level of nature connectedness and a decrease in the level of pro-environment behavior, while the nature connectedness can affect the level of pro-environment behavior. Our research hypothesis was as follows: the mediating effect of nature connectedness on the relationship between materialistic values and pro-environmental behavior (H2).

## Natural Contact Paradigm and Nature Connectedness

Nowadays, young people clearly encounter an extraordinary array of vicarious images of nature. Young people's experience of nature, broadly speaking, can be classified in three ways: direct, indirect, and what may be called “vicarious” or “symbolic” experience (Kellert, 2002). For the majority of people today, outdoor nature experiences, while vanishing, can be replaced with virtual alternatives (Clements, 2004; Ballouard et al., 2011). These vicarious images often occur in modern society through relatively innovative communication technologies like mobile phones, television, film, or computers (Kellert, 2002). The environmental connectedness perspective also posits that direct encounters with generalized or non-specific “nature” lead to environmental connectedness (Kellert, 2002; Beery and Wolf-Watz, 2014). There is evidence that the longer the contact time with nature, the stronger the sense of connection with nature (Kals et al., 1999; Chawla, 2006). In addition, some studies have shown that after viewing pictures or videos of nature, known as the nature exposure paradigm, individuals scored higher on nature connectedness (Weinstein et al., 2009; Zelenski et al., 2015; Soga et al., 2016). Importantly, there is evidence that four laboratory studies (where participants reported their wishes after having been exposed to a particular scene or object) showing that individuals focused on their internal aspirations more than on external ones when they were exposed to nature stimuli (Weinstein et al., 2009). It may be that when people are guided to focus on intrinsic and self-transcendent goals, they move away from materialistic values. Taken together, we hypothesized the following: enhancing the nature connectedness through natural contact paradigm could mitigate the impact of high materialism on pro-environment behavior (H3).

## The Present Study

To extend the former research, three main points have been improved in this study. First, at present, the evidence that high materialistic values are predictive of lower pro-environmental behavior among youth and young adults is scant and relatively weak especially in a Chinese cultural context (Gu et al., 2020). Most such research is cross-sectional. Moreover, such research has not examined the underlying psychological mechanisms between materialistic values and pro-environment behavior. Third, Kasser (2016) suggests that while acknowledging that everyone has materialistic values or goals, proposed ways to reduce the damage of materialism to other valuable life goals. As for how to promote the possibility of pro-environment behavior of people with materialistic values, this is also a problem worthy of attention and discussion.

Accordingly, considering the limitations of previous studies, we conducted a questionnaire survey (Study 1) and an experimental study (Study 2) to examine the causal relationship between materialism and pro-environmental behavior, and to verify the intermediary role of nature connectedness in the relationship. Further, to thoroughly examine whether the effects of high levels of materialism on pro-environment behavior can be mitigated, Study 3 used the natural contact paradigm to manipulate the level of nature connectedness to further test the role of the nature connectedness in the relationship between high materialistic values and pro-environmental behavior.

## Study 1

### Participants and Procedure

A power analysis using G^*^Power software (Faul et al., 2009) analysis suggested that to obtain a medium power test (*r* = 0.30, α = 0.05, 1-β = 0.80), 82 participants were needed. Considering that questionnaires attrition rate may be low, we recruited 305 students from two local universities in Xi'an, China, to complete a questionnaire, and 277 questionnaires were collected (recovery rate = 90.8%). Participants (119 women) were between 18 and 24 years of age (*M*_age_ = 20.85 years, *SD* = 1.53). Participants were informed about the nature of their participation in the study and provided their verbally consent to participate. After completing the questionnaire, each participant was given a pen in appreciation.

### Measures

#### Trait Materialistic Values

The revised Chinese version of the Material Values Scale (MVS; Li and Guo, 2009), originally developed by Richins and Dawson (1992), was used to measure trait materialism. The structure of the revised scale is similar to that of the original scale; however, five items were removed due to cultural differences and translation problems (Li and Guo, 2009). This is a reliable and valid measurement for assessing trait materialism in Chinese populations (Li et al., 2018) and includes 13 items rated on a five-point Likert scale (1 = “strongly disagree,” 5 = “strongly agree”). The reliability of the scale in the present study was Cronbach's α = 0.78.

#### Pro-environmental Behavior

We used the self-report Pro-environmental Behavior Scale (PBS), revised by Liu and Wu (2013) for Chinese college students in reference to past research (Kaiser et al., 2007; Gong, 2008). This scale consists of 12 items, and participants rate each item from one to five according to their actual behaviors. The total score ranges from 12 to 60. The PBS includes items such as “I am actively involved in activities organized by the school or the environmental protection society” and “When no one is in the room, I will turn off the lights when I leave the room.” Higher scores on the PBS indicate higher pro-environmental behavior. In previous studies, the reliability and validity of the questionnaire were good, and the Cronbach α coefficient was above 0.78 (Zong and Wang, 2017). In the present study, Cronbach's α for the PBS was 0.81.

#### Nature Connectedness

We used the Connectedness to Nature Scale (CNS) developed by Mayer and Frantz (2004) and revised by Li and Wu (2016). Participants rate their agreement with 14 items using a five-point scale (1 = “strongly disagree,” 5 = “strongly agree”); items 4, 12, and 14 are reverse-scored. The scale is unidimensional, and higher total scores indicate higher emotional connection and a closer relationship with nature. The CNS has been found to be reliable across time and is widely used (Diebels and Leary, 2019). The reliability of the scale in our study was Cronbach's α = 0.78.

### Results and Discussion

#### Common Method Biases

According to the suggestion of Podsakoff et al. (2003), the Harman single-factor test used to test common method deviation. That is, an unrotated principal component factor analysis is performed on all variables simultaneously. If multiple factors are obtained and the variation explained by the first factor does not exceed 40%, the common method variation problem is not serious. The results revealed 11 eigenvalues >1 without rotation, and the mutation rate interpretation of the first factor was 15.94%, which was less than the critical value of 40%. This indicated that the common method deviation in this study was not problematic, and subsequent data analysis could be carried out.

#### Descriptive Statistics and Correlations

We computed Pearson's correlation coefficients to explore the relationships among the study variables (see Table 1 for the descriptive statistics and correlations). The results showed that materialistic values had a significant negative correlation with pro-environmental behavior. Nature connectedness showed a significant negative correlation with materialistic values and a significant positive correlation with pro-environmental behavior.

**Table 1:** Descriptive statistics and correlation coefficients for materialistic values, nature connectedness, and pro-environmental behavior.

	***M***	***SD***	**1**	**2**	**3**
1. Materialistic values	2.56	0.58	–		
2. Nature connectedness	3.89	0.57	−0.29^**^	–	
3. Pro-environmental behavior	3.52	0.60	−0.12^*^	0.28^**^	–

*Sample size = 277;*

* *p < 0.05;*

** *p < 0.01*.

#### Mediation Analysis: The Role of Nature Connectedness

**Figure 1:**
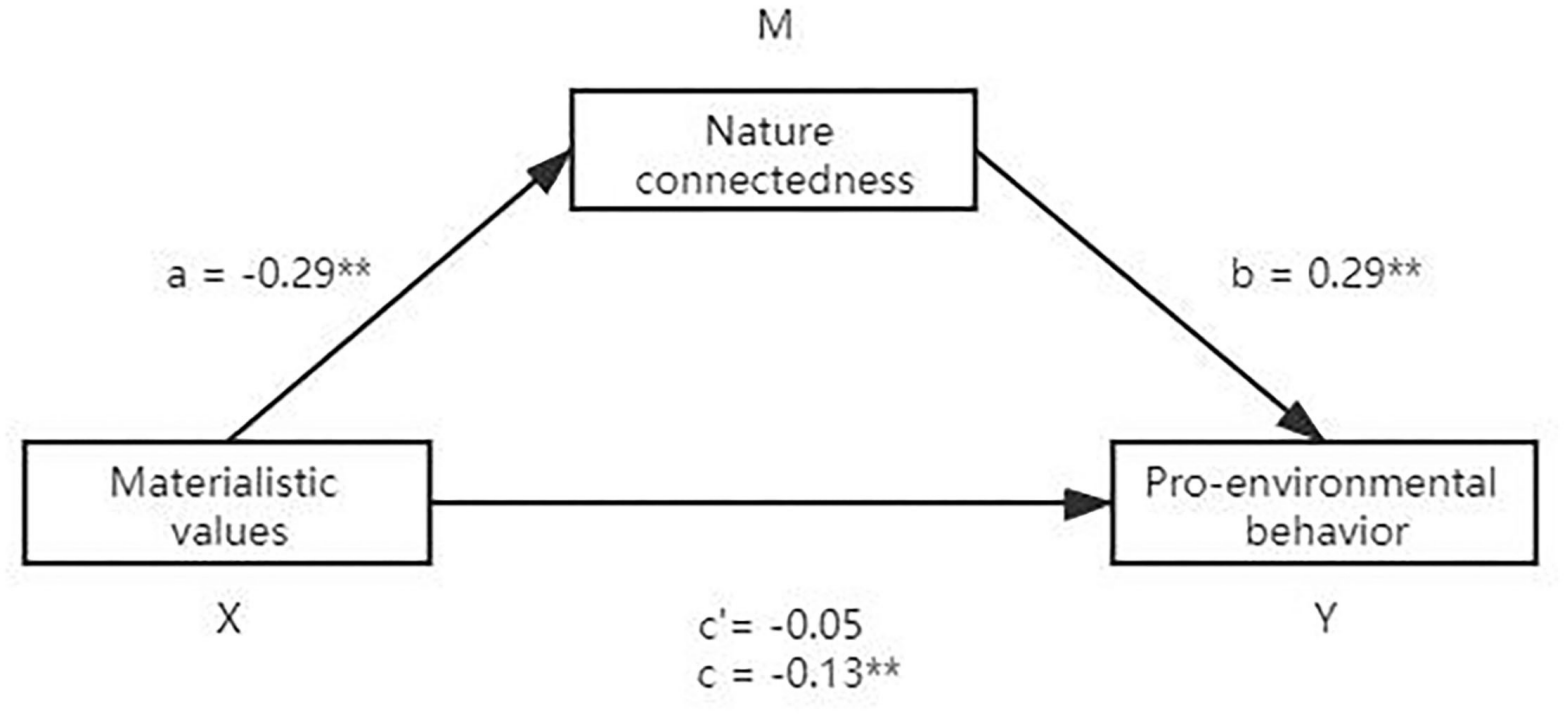
Mediating effect of nature-connectedness on the relationship between materialistic values and pro-environmental behavior.

We examined the mediating role of nature connectedness in the association between materialistic values and pro-environmental behavior by employing the PROCESS 3.0 (Hayes, 2013) macro (Model 4, 5,000 bootstrap samples) of SPSS. Materialistic values was the independent variable (X), pro-environmental behavior was the dependent variable (Y), and nature connectedness was the mediating variable (M). The results are shown in Figure 1. In the direct path (c), materialistic values negatively predicted pro-environmental behavior. In the indirect path, materialistic values negatively predicted nature connectedness (a), while nature connectedness positively predicted pro-environmental behavior (b). The mediating effect value of nature connectedness between materialistic values and pro-environmental behavior was -0.08, 95% confidence interval (CI) [-0.1405, -0.0349], and this range did not include zero. The ratio of indirect to direct effect of materialistic values on pro-environmental behavior was 39%. Therefore, the mediating mechanism of nature connectedness had statistical significance in the relationship between materialistic values and pro-environmental behavior.

In summary, we used a cross-sectional questionnaire to explore the relationship between materialistic values and pro-environmental behavior, and the mediating role of nature connectedness. Results showed that the level of pro-environmental behavior of participants with high materialistic values was lower compared with participants with low materialistic values. Low pro-environmental behavior was often accompanied by a high level of materialistic tendencies and low levels of nature connectedness. This result provides support for our hypothesis but does not explain the causal relationships. Hence, Study 2 was performed to further explore the relationships among the three variables through experimental manipulation of materialism.

## Study 2

### Participants

To obtain a medium power test (effect size = 0.55 in a *t*-test analysis), a G^*^power analysis suggested a total sample size of 106 participants would be needed to obtain a power of 0.80 (Faul et al., 2009). Therefore, we recruited 140 college students from two local universities in Xi'an, China; data from 3 participants were removed because they did not complete the experiment as required. Thus, 137 participants (68 women) aged between 18 and 24 years (*M*_age_ = 18.88 years, *SD* = 1.07) completed the experiment. Participants provided their informed consent to participate.

### Stimulus, Materials, and Procedure

#### Materialistic Values Manipulation

We referred to the activation theory of materialistic values and previous research (Bauer et al., 2012; Caruso et al., 2013) and selected cues reflecting materialism in daily life such as cars, brand-name cosmetics, jewelry, and luxury goods (30 pictures from an Internet gallery). We also selected 30 geometric pictures (including planar, three-dimensional regular or irregular) to be used in the neutral control group in the same way, according to Berry et al. (2014) study. These images were randomly presented to the experimental and control groups, respectively (rendering time: 30,000 ms; interval: 5,000 ms).

#### State Materialistic Values Scale

We modified the Chinese version of the MVS (Li and Guo, 2009) used in Study 1 to develop a state materialism scale by adding “At the moment,” so that items referred to the participants' current state of mind (e.g., “At the moment, I think I will feel happier if I can afford to buy more things”). The scoring criteria were the same as in the original scale. The reliability of this revised scale in our study was Cronbach's α = 0.73.

#### State Connectedness to Nature Scale

The state CNS version contained 13 of the 14 items in the trait CNS version (Study 1) simply reworded. The state CNS was well-correlated with the trait CNS (*r* > 0.6) and has been successfully used to measure connectedness to nature due to experimental manipulations in previous studies (Frantz et al., 2005). For example, the item, “I often feel a sense of oneness with the natural world around me” was rephrased as “At this moment I feel a sense of oneness with the natural world around me.” One item from the original scale was deleted because it could not be reworded. Items were rated on a five-point Likert scale (1 = “strongly disagree,” 5 = “strongly agree”). The reliability of this scale in our study was Cronbach's α = 0.76.

#### Pro-environmental Behavior Scenario Simulation Task

We used a pro-environmental behavior scenario simulation task. The level of pro-environmental behavior was measured by assessing simulated donations to an environmentally friendly organization (Ku and Zaroff, 2014; Zaval et al., 2015), and participants received the following information. “Our research group is cooperating with the environmental protection organization of our school *Friends of Green* to carry out the environmental protection activity of urban garbage reduction. This activity aims to explore new models of waste disposal and provides support for relevant policy formulation and public knowledge popularization. Many a little makes a mickle. You can choose to convert this gift into 10 RMB. We hope you can take some money from this experiment remuneration to help our environmental protection activities. If the fee is divided into 10 parts, from 0 to 10, how many parts of the community chest would you donate to help the environmental campaign?”

### Procedure

After the participants entered the laboratory, we asked them to sign an informed consent form, and explained to them that the experimental procedure was divided into three parts. Then, the participants were each randomly allocated to one of two conditions: materialism condition (*n* = 69) and neutral priming condition (*n* = 68). Initially, participants completed a photo categorization game (priming task). The priming group was shown materialism-inducing material, and the control group was shown the geometric material. Immediately afterwards, they were asked to complete the measures of state materialism and state nature connectedness. Next, participants were asked to complete the pro-environmental behavior scenario simulation task and a demographic questionnaire. Participants were informed that the survey was anonymous and confidential and were asked to answer truthfully. After the experiment, each participant was debriefed to determine their awareness of the study hypotheses, and no one was able to identify the study's true purpose. Participants were given a notebook as a token of appreciation.

### Results and Discussion

#### Materialistic Values Manipulation Check

We compared the state materialistic values scores of the priming group with the control group to check the manipulation of materialism. The analysis revealed that the state materialism values scores in the priming group (*M* = 3.52, *SD* = 0.46) were significantly higher than in the control group (*M* = 2.84, *SD* = 0.46), *t*(68) = −8.64, *p* < 0.001, which suggests that the manipulation of state materialism was successful.

#### Pro-environmental Behavior

We compared the money donated to the environmental organization the number between the materialism priming group and control group to test the influence of materialistic values on pro-environmental behavior. The results showed a significant difference in the scores of the two groups. Participants in the materialism priming group (*M* = 5.62, *SD* = 2.21) chose to donate significantly fewer money for environmentally friendly activity than those in the control group (*M* = 8.21, *SD* = 1.53), *t*(68) = 7.94, *p* < 0.001. In addition, The indices indicated that participants who had temporarily enhanced materialism in the laboratory showed lower levels of pro-environmental behavior.

#### Mediation Analysis: The Role of Nature Connectedness

The correlations between the study variables are shown in Table 2. There were significant correlations between materialistic values, nature connectedness, and pro-environmental behavior, which provided a basis for testing the mediating effect.

**Table 2:** Descriptive statistics and intercorrelations between variables in Study 2.

	***M***	***SD***	**1**	**2**	**3**
1. Materialistic values	3.18	0.57	–		
2. Nature connectedness	2.84	0.50	−0.47^**^	–	
3. Pro-environmental behavior	6.91	2.30	−0.39^**^	0.42^**^	–

** *p < 0.01*.

Next, we used the PROCESS macro of SPSS 23.0 (Model 4, 5,000 bootstrap samples) to estimate the mediating effect of nature connectedness on the relationship between materialistic values and pro-environmental behavior. Results revealed that the materialistic values negatively predicted nature connectedness (β = −0.41, *p* < 0.001), which in turn negatively predicted the score for number of green products purchased (β = −1.01, *p* < 0.005). The residual direct effect was also significant (β = −1.01, *p* < 0.005). The total effect of materialism on the score for number of green products purchased was significant (total effect = −1.58, 95% CI = [-2.21, -0.95]). After nature connectedness was entered into the equation, the indirect negative effect of materialism on the pro-environmental behavior remained significant (indirect effect = −0.57, 95% CI = [-1.01, -0.20]). The results supported the role of nature connectedness as a partial mediator.

The results from Study 2 conceptually replicated Study 1 (by manipulating the individual relative level of materialistic tendency) and provided support for the hypothesis. As a special case of pro-social behavior, pro-environmental behavior has a high degree of social desirability. According to a meta-analysis performed Kormos and Gifford (2014), self-reported pro-environmental behavior can only explain 21% of the variance in actual behavior. Therefore, to improve the ecological validity of the research, this study adopted a simulation of giving money to environmental groups. We found that participants with higher materialism tended to show lower levels of pro-environmental behavior due to their lower nature connectedness. If nature connectedness is an intervention strategy with negative effects on materialism, it should increase the level of pro-environmental behavior among highly materialistic individuals. Therefore, in Study 3, we explored whether the activation of nature connectedness could enhance the pro-environmental behavior of high-materialism participants.

## Study 3

### Participants

Referring to the sample size of study 2 using G^*^power analysis and considering the needs of this study, we recruited 420 college students from two local universities in Xi'an, China, who had not participated in the previous experiment, then completed the MVS questionnaire. Data from 17 participants were excluded due to incomplete information (recovery rate = 95.95%). Total MVS scores were ranked from low to high. The highest 27% were selected as the high-materialism group. Thus, the participants were 109 college students between 18 and 27 years of age (58 women; *M*_age_ = 19.38 years, *SD* = 1.70). Participants provided their informed consent to participate.

### Stimulus, Materials, and Procedure

#### State Materialistic Values Scale

The measure of trait MVS was the same as in Study 1, and Cronbach's α was 0.86.

#### State Connectedness to Nature Scale

The measure of state CNS was the same as in Study 2, and Cronbach's α was 0.78.

#### Natural Contact Paradigm

Referring to a prior study using nature-contact materials (Mayer et al., 2009), we selected 5 × 3-min videos from network documentaries of Chinese nature scenes. We asked five doctoral students in psychology to establish corresponding scores for these clips using the “structural evaluation of the environment” method (Ulrich et al., 1991) and different dimensions (complexity, structure, concerns, and scene depth). After extensive deliberation, we selected the video with high scores in all aspects as the material for the nature group. The control group procedure was consistent with the method in Study 2; as such, we selected videos of geometric shapes ~3 min in length.

#### Pro-environmental Behavior Scenario Simulation Task

We added a score on willingness to donate time to environmental activities from study 2. After completing the money donation option, participants were given the following message: “If you divide your leisure time into 10 parts, from 0 to 10, how much time do you have for environmental activities?”

### Procedure

As in Study 2, after providing informed consent, participants were randomly divided into two groups: nature exposure (*n* = 55) and neutral control (*n* = 54). First, participants were required to watch videos with nature content or geometric figures, depending on their group assignment, and then complete the state CNS and a demographic questionnaire. Following this, participants were asked to complete the second task: making a donation of money and time to the environmental organization. We assured them that the survey was anonymous and confidential, and asked them to answer truthfully. After the experiment, each participant was debriefed to determine their awareness of the study hypotheses, and no one was able to identify the study's true purpose. Participants were given a notebook in appreciation.

### Results and Discussion

#### Nature Connectedness

To test the level of nature connectedness between the nature exposure and neutral control, we used *T*-test to compared the sense of nature connectedness in the two groups.

The analysis revealed that the mean state CNS score in the nature exposure group (*M* = 3.91, *SD* = 0.43) was significantly higher compared to the control group (*M* = 3.41, *SD* = 0.53), [*t*(95) = 5.43, *p* < 0.001]. These results suggest that natural contact intervention strategies can effectively enhance the sense of natural connection inhibited by high materialistic values.

#### Descriptive Statistics and Correlations

Descriptive statistics were used for participants' general characteristics with high materialistic values and descriptive statistics of the measurement variables (see Table 3). The results showed that materialistic values had a significant negative correlation with nature connectedness and money donated to environmental organization, except time donated to environmental organization. Nature connectedness showed a significant positive correlation with money donated to environmental organization and time donated to environmental organization. Meanwhile, there is a strong positive relationship between the money donated to environmental organization and time donated to environmental organization.

**Table 3:** Descriptive statistics and intercorrelations between variables in Study 3.

	***M***	***SD***	**1**	**2**	**3**	**4**
1. Materialistic values	4.09	0.28	–			
2. Nature connectedness	3.66	0.54	−0.24^*^	–		
3. Money donated to environmental organization	6.04	2.53	−0.21^*^	0.22^*^	–	
4. Time donated to environmental organization	5.53	2.65	−0.17	0.21^*^	0.45^**^	–

* *p < 0.05;*

** *p < 0.01*.

#### Comparison of Pro-environmental Behavior at Different Levels of Nature Exposure

To assess the effect of the nature contact intervention on pro-environmental behavior among individuals with high materialistic tendencies, a *t*-test was conducted to compare the money and time donated to the environmental organization between the nature exposure and control groups. The results showed a significant difference between the two groups (Table 4); specifically, participants in the nature exposure group willing to donate more money to the environmental organization and spend more time in environmental activities than those in the control group.

**Table 4:** Comparison of pro-environmental behavior at different levels of nature exposure.

**Group**	***n***	**Money donated to environmental organization**				**Time donated to environmental organization**			
		***M***	***SD***	***t***	***p***	***M***	***SD***	***t***	***p***
Nature exposure group	55	7.27	2.30	5.91	0.000	6.36	2.72	3.47	0.001
Control group	54	4.78	2.12			4.69	2.30		

Therefore, we believe that the natural contact paradigm can effectively enhance the emotional relationship of individuals with materialistic values to the environment. Nature contact paradigm could enhance pro-environmental behavior for individuals with high materialistic tendencies in a short period of time. The negative effect of high materialistic tendencies on pro-environmental behavior may decrease or disappear with increased contact with nature, leading to an increase in nature connectedness. Therefore, the covariant relationship between materialistic values, nature connectedness, and pro-environmental behavior was further verified.

## General Discussion

We designed three studies to investigate the influence of materialism on pro-environmental behavior and the underlying psychological mechanisms. Studies supported our prediction that individuals with higher materialism tendencies would have a lower level of pro-environmental behavior, and that nature connectedness would play an intermediary role in this relationship. Moreover, study 3 also demonstrated natural contact paradigm can effectively enhance the sense of nature connection and the level of pro-environmental behavior among those with high materialism.

### Relationship Between Materialism and Pro-environmental Behavior

Study 1 and 2 both showed that materialistic values negatively predicted pro-environment behavior. Compared with participants withe low levels of materialistic values, those with high levels of materialistic values reported fewer pro-environmental behaviors. These results were consistent with previous studies. For example, researches in different areas have shown that college students with high materialism are less likely to consider environmental protection to be a goal for their future behavior (Hirsh and Dolderman, 2007; Hultman et al., 2015; Lu et al., 2016). China has developed a largely materialistic culture, marked by the pursuit of pretention, identity, and status. Thus, there is a need to examine the relationship between individual materialistic tendencies and pro-environmental behavior in the context of the local culture in China.

### Psychological Mechanisms of How Materialism Influences Pro-environmental Behavior

In a previous study by Soga and Gaston (2016), the results showed that the individual's development in industrial social environments was highly separated from nature, and the loss of direct or indirect nature experience may reduce individuals' positive emotions toward nature and willingness to protect it. Recently, Whitburn et al. (2020) found that indicators related to nature connectedness were significantly positively correlated with pro-environmental attitudes and behavior. These studies confirm the important role of connection to nature in environmental sustainability (Zelenski and Nisbet, 2014). Consistent with these previous studies, our findings showed that the negative effect of high materialism on pro-environmental behavior may be due to the suppression of nature bonding emotions.

It is not sufficient to only demonstrate the mediating role of nature connectedness, because we could merely speculate that high materialists exhibit lower pro-environmental behavior due to the inhibition of their nature connectedness. To address this issue, we directly verified the mitigating effect in Study 3. Individual contact with nature is regarded as a key factor in cultivating a good relationship with nature (Chawla, 2006). Although there is a difference between actual and virtual experiences of nature, studies have often shown that nature simulation can replace actual nature (Kellert, 2002; Beery and Wolf-Watz, 2014). The frequency of viewing nature-themed videos and books was found to be positively correlated with children's emotional attitudes toward local biodiversity, promoting a stronger sense of connection between people and nature (Soga et al., 2016). Our research also confirmed this result. In addition, we found that improving one's connection to nature was a successful pathway to mitigating the negative effects of high levels of materialism. Our results are consistent with Weinstein et al. (2009), individuals of high materialistic values with higher levels of affiliation with nature report greater engagement in ecological behavior, and willingness to display pro-environmental behavior.

The present study contributes to the literature in several ways. First, it extends our understanding of the relationship between materialistic values and pro-environmental behavior. Previous studies (Wierzbicki and Zawadzka, 2016) have focused only slightly on the relevant psychological mechanisms, which was not conducive to explaining the underlying causes of materialism's influence on pro-environmental behavior. Therefore, our research provided the initial evidence on the intermediary role of nature connectedness in the relationship between materialism and pro-environmental behavior. Individuals with materialistic goals, such as pursuing status and financial success, tend to be more prone to conspicuous consumption and accumulation of high-status commodities (Liao and Wang, 2009). This suggests that, to an extent, an individual's materialism-based dreams have a greater negative impact on the environment. This new finding not only helps construct new models for this relationship but also contributes to developing new approaches to intervene in it.

The findings of our research also have significant practical implications. Studies have shown that enhancing individuals' sense of nature connectedness can affect the relationship between materialism and environmentally friendly behavior, which could play an important role in further improving environmentally friendly activities. This is conducive to helping individuals find a balance between the great enrichment of material wealth and the sustainable development of the ecological environment, thus reducing the social risks for the benefit of future generations. Specifically, whether it is the government's social governance or enterprises' product promotion, indirect or direct nature contact in various forms can be increased, so as to promote a sense of connection with nature to the greatest extent and thereby increase environmentally friendly behavior.

## Limitations and Future Research Directions

Limitations to this research need to be addressed by future studies. First, this research only selected college students as participants, which limits the generalizability of our results. Moreover, the display of materialism across different life stages is not completely consistent (Jaspers and Pieters, 2016). Therefore, future research should test our hypotheses with a broader range of participants of all ages from the general population. Second, our studies only examined the mediating role of nature connection. However, the relationship between materialism and pro-environmental behavior is complex and diverse (Kollmuss and Agyeman, 2002). To clarify the action mechanism between these constructs and identify the strongest influences, other variables can be studied in depth in the future to promote pro-environmental behavior and improve environmental quality. Third, the present study demonstrated that the sense of nature connectedness enhanced environmental behavior in individuals with high materialistic tendencies in a short time, but other long-term effective means of weakening this relationship should be explored.

## Data Availability Statement

The original contributions presented in the study are included in the article/Supplementary Material, further inquiries can be directed to the corresponding author/s.

## Ethics Statement

The studies involving human participants were reviewed and approved by Experimental Research Ethics Review Committee, School of Psychology, Shaanxi Normal University. The patients/participants provided their written informed consent to participate in this study.

## Author Contributions

JW: conceptualization, software, and writing—original draft. YH: supervision and validation. Both authors contributed to the article and approved the submitted version.

## Funding

This work was supported by the Fundamental Research Funds for the Central Universities (grant number 2019TS139).

## Conflict of Interest

The authors declare that the research was conducted in the absence of any commercial or financial relationships that could be construed as a potential conflict of interest.

## Publisher's Note

All claims expressed in this article are solely those of the authors and do not necessarily represent those of their affiliated organizations, or those of the publisher, the editors and the reviewers. Any product that may be evaluated in this article, or claim that may be made by its manufacturer, is not guaranteed or endorsed by the publisher.
